# Harmonic Generation in Biased Semiconductor Superlattices

**DOI:** 10.3390/nano12091504

**Published:** 2022-04-28

**Authors:** Mauro Fernandes Pereira

**Affiliations:** 1 Department of Physics, Khalifa University of Science and Technology, Abu Dhabi 127788, United Arab Emirates; mauro.pereira@ku.ac.ae; 2 Institute of Physics, Czech Academy of Sciences, 18221 Prague, Czech Republic

**Keywords:** gigahertz, terahertz, sub-terahertz, semiconductor superlattices, harmonic generation

## Abstract

Semiconductor superlattices are proven nanomaterials for THz nonlinear optics by means of high order harmonic generation. Seminal approaches leading to a perfectly antisymmetric current-voltage (I–V.) curve predict the generation of odd harmonics only in the absence of a bias. However, even harmonics at high orders have been detected in several experiments. Their generation has been explained by considering deviations from the current flow symmetry that break the exact antisymmetry of the I–V. curve. In this paper, we focus on another issue found experimentally that has also not been explained, namely the harmonic power output asymmetry from negative to positive applied bias. Once more, breaking the I–V. flow symmetry explains the experiments and leads to a further tool to design the power output of these materials. Furthermore, a new approach for the Boltzmann Equation under relaxation-rate approximation eliminates numerical difficulties generated by a previous theory. This leads to very efficient analytical expressions that can be used for both fundamental physics/optics/material sciences and realistic device development and simulations.

## 1. Introduction

Sub-Terahertz and Terahertz (THz) photonics are topics of increasing interest for a wealth of different applications, fueled by the availability of new materials, sources, and detectors [1,2,3,4,5,6]. Among these materials, semiconductor superlattices (SSLs), which display a periodic structure and are grown by epitaxial growth techniques, offer enormous flexibility to control quantum transport and optics phenomena [7,8,9,10,11,12,13]. Together with quasi-periodic structures, they play a major role in controllable nonlinear optical effects, such as High Order Harmonic Generation (HHG) all the way to the 54th harmonic at 37 µm. Superlattice multipliers (SSLMs) are efficient as both sources and heterodyne detectors [14,15]. The main competing technology for SSLMs is quantum cascade lasers [16] and detectors, whose wafers are also generated by epitaxial growth methods and can likewise be simulated and designed with techniques similar to those that have been developed to study quantum cascade lasers [17,18,19,20,21,22,23,24,25,26,27,28,29,30,31].

**Figure 1:**
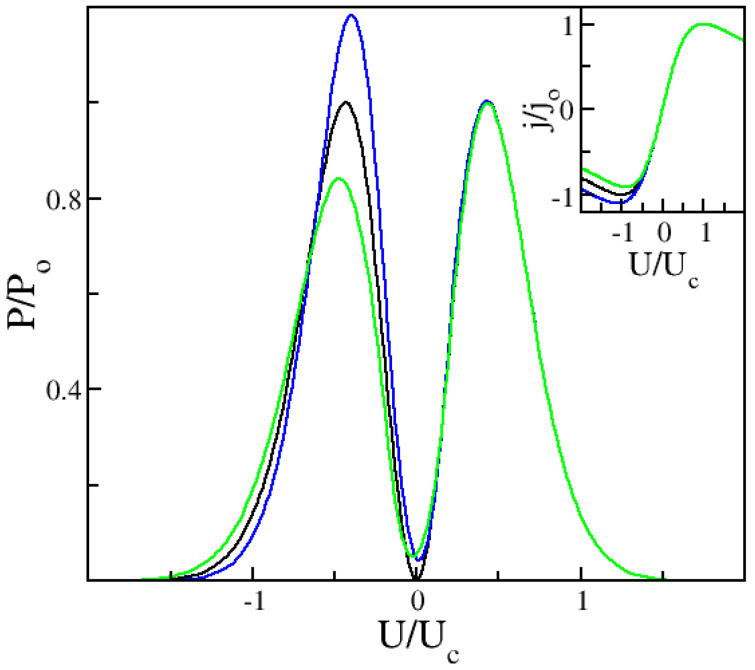
Second Harmonic power output dependence on the current flow asymmetry, ruled by the parameter *δ*. The inset depicts the corresponding I–V. The black line is for *δ* = 1, i.e., symmetric current flow leading to perfectly antisymmetric I–V. The blue and green curves are, respectively, for *δ* = 1.1 and *δ* = 0.91. The input frequency is *ν* = 120 GHz and power control parameter is *α* = 20. J_0_, P_0_, and U_c_ are, respectively, the peak current and peak output. The critical voltage at *δ* = 1 is given by U_c_. It determines the region of negative differential conductivity.

Electrons in an SSL are accelerated under the influence of a static field of amplitude $E_{dc}$. Since SSLs are characterized by a tight-binding-like dispersion relation, this directly leads to oscillations in velocity and spatial position, with a frequency given by $\nu_{B}=eE_{dc}d/h$. These oscillations are called Bloch oscillations and they are responsible for the strong nonlinear current-voltage characteristic of SSLs, as shown in Figure 1. Esaki and Tsu proposed the application of a high-frequency field to the SSLs. For a review, see Ref. [7].

The demonstration of coherent Bloch oscillations paved the way for the development of efficient SSLMs [32]. An oscillating electromagnetic field can modulate the Bloch oscillations in an SSL giving rise to HHG [33,34,35,36]. Electric field domains can have a strong impact on this process [37,38,39], as well as excitonic effects [40,41,42]. Here, we focus on effects that develop when SSLMs with a current flow that is intrinsically asymmetric with respect to an applied negative or positive bias, using a modulated Bloch oscillation model [7,8,9,29,30,31,43,44,45,46,47].

Note, however, that there are other very interesting materials and processes photonics applications in the THz range. For example, Bragg-spaced quantum wells BSQWs can be used to produce tunable slow light delays [48]. Furthermore, Terahertz Spin Currents have been generated and controlled in Topology-Induced 2D Ferromagnetic Fe_3_GeTe_2_|Bi_2_Te_3_ heterostructures [49] which are very different from the heterostructures considered here, showing that many different materials can lead to important developments in the THz range. Also of relevance is the fact that here we focus on harmonic generation inside the heterostructure. The role played by the interfaces is to break the symmetry in current flow leading to high order even harmonic generation. In contrast, second harmonic generation has been demonstrated in AB-type LaTiO_3_/SrTiO_3_ superlattices [50] where surfaces and interfaces play a major role in the nonlinear process. Note, however, that this is a second-order $\chi^{\;\left(2\right)}$ nonlinear susceptibility effect and that in our SSLMS, we exploit a type of nonlinearity that cannot be explained with conventional $\chi {\;}^{\left(n\right)}$ nonlinear susceptibilities, as demonstrated theoretically and experimentally in Ref. [47].

## 2. Materials and Methods

Previous studies of HHG were based on a theory that takes input from a Nonequilibrium Green’s Functions (NEGF) approach for the current-voltage. It calculates the HHG that develops in a biased superlattice when an oscillating field modulates the Bloch oscillations. This approach allowed the prediction and measurement of control of even harmonics by orders of magnitude [47]. The NEGF input is used in combination with the Boltzmann equation and the HHG is obtained from the nonlinear oscillating current. There is an exact solution for symmetric current flow and two approaches for the non-symmetric flow case that explains the even harmonics in good agreement with experiments: an Ansatz solution that leads to analytical solutions [29] and a path integral technique for non-perturbative solutions [30]. The analytical Ansatz is attractive for its programmable simplicity but leads to numerical problems for low powers of the oscillating field. Here, we introduce a better Ansatz, which is fully differentiable at zero voltage in contrast to the previous approach. It delivers a numerical-error-free set of analytical expressions. A miniband transport model [7,29,30,31,43,44,45,46,47] for the Boltzmann Equation in the relaxation rate approximation, leads to a nonlinear current density. This model can be solved for an arbitrary dependence in an applied electric field,

(1) $$j\left(t\right)=2j_{0}\int_{-\infty }^{t}dt_{1}e^{-\left(t-t_{1}\right)\Gamma /\hbar }sin\left[\int_{t_{1}}^{t}dt_{2}\frac{eE\left(t_{2}\right)d}{\hbar }\right]$$

The results that we wish to analyze, require exposure of the SSLM to a combined static bias and a monochromatic field, $E=E_{dc}+E_{ac}\cos\left(2\pi \nu t\right).$ Direct substitution of this field into Equation (1) and a time average, developed in detail in Refs. [29,30,31,43,44,45,46,47], lead to the following expression for the electromagnetic power emitted by the nth harmonic

(2) $$P_{n}=𝒫\left[j_{n,s}^{2}\;+\;j_{n,c}^{2}\right],$$

(3) $$\;j_{n,s}=\overset{\infty }{\underset{m=-\infty }{\sum }}J_{m}\left(\alpha \right)\left[J_{m+n}\left(a\right)-J_{m-n}\left(a\right)\right]K\left(U\right)$$

$$j_{n,c}=\overset{\infty }{\underset{m=-\infty }{\sum }}J_{m}\left(\alpha \right)\left[J_{m+n}\left(a\right)+J_{m-n}\left(a\right)\right]Y\left(U\right)$$

**Figure 2:**
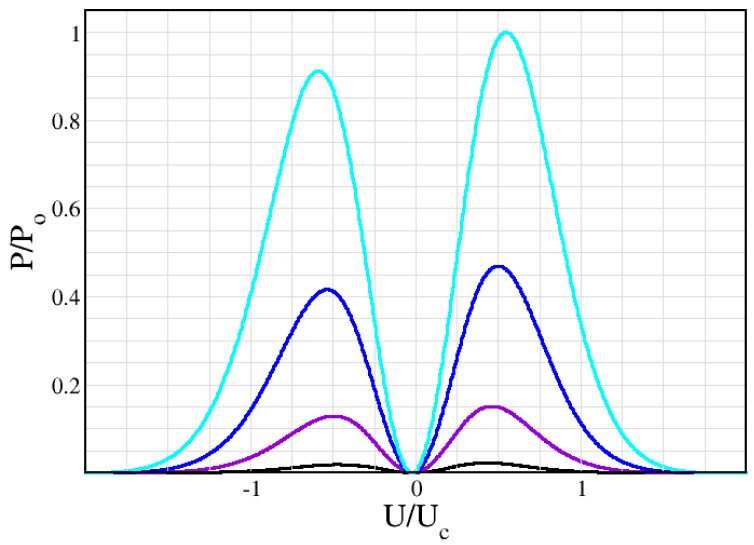
Second Harmonic power output for different values of the power control parameter, with an input frequency *ν* = 120 GHz. From bottom to top, *α* = 10, 16.7, 23.3 and 30, corresponding to black, violet, blue and cyan curves. The current flow asymmetry for all curves is *δ* = 0.91. U_c_ is the same as in Figure 1 and P_0_ is the maximum for α = 30.

**Figure 3:**
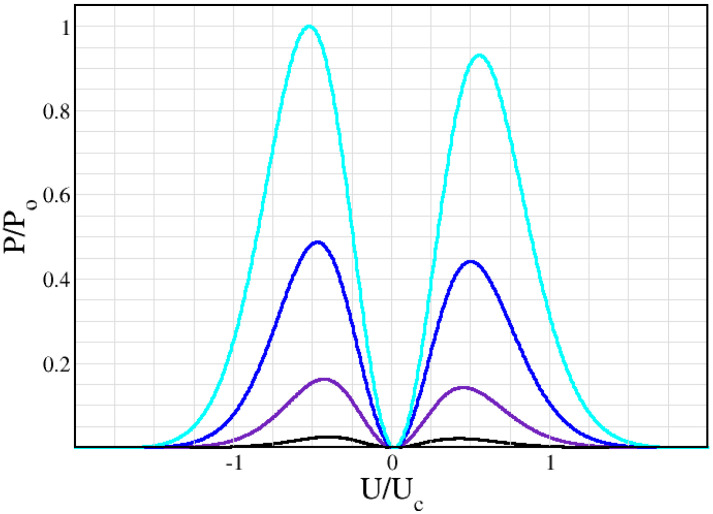
Second Harmonic power output for different values of the power control parameter, with an input frequency *ν* = 120 GHz. From bottom to top, *α* = 10, 16.7, 23.3 and 30, corresponding to black, violet, blue and cyan curves. The current flow asymmetry for all curves is *δ* = 1.1. U_c_ is the same as in Figure 1 and P_0_ is the maximum for *α* = 30.

**Figure 4:**
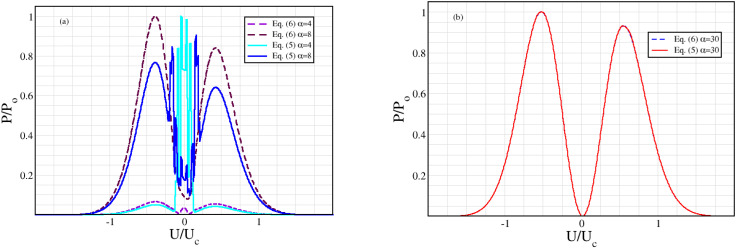
Comparison between the Second Harmonic power output, with an input frequency *ν* = 120 GHz using either Equation (5), shown as solid curves, or Equation (6), shown as dashed curves. In panel (**a**) For Equation (5) *α* = 4 in cyan and *α* = 8 in blue. For Equation (6), *α* = 4 in violet and *α* = 8 in maroon. In panel (**b**) *α* = 30: dashed blue for Equation (6) and solid red for Equation (5). The current flow asymmetry for all curves is *δ* = 1.1. U_c_ is the same as in Figure 1. There is almost no difference between both approaches for large *α* and a large difference for very small *α*. The calculations presented here are consistent with the experiments in Ref. [47].

Here, 𝒫 is a numerical constant derived in detail in Ref. [30] and $J_{m}\;$are Bessel functions of the first kind and order m. The parameter $\alpha =eE_{ac}d/\left(h\nu \right)$, appears naturally from the formalism and depends on the amplitude of the oscillating field. Equation (3) clearly shows that α rules the modulation degree of Bloch frequency oscillations through the factors depending on $J_{m}\;\left(\alpha \right),\;J_{m\;\pm \;n}\;\left(\alpha \right)$. Therefore, α effectively controls the nonlinear response of the system [12]. Evidence of this modulation is given in Figure 1, Figure 2, Figure 3 and Figure 4 of this paper, namely thought studies of the harmonics, which have been studied experimentally in Ref. [33]. A theoretical analysis of the buildup and modulation of harmonics is given in Ref. [31]. The rectified potential drop is $U=eE_{dc}d+mh\nu$. This affects even the static I–V. leading to a very interesting current rectification by photon-assisted tunneling that has been confirmed by experiments [7,47]. The superlattice period, Plank’s constant and the electron charge are given, respectively, by d,h,e. Y and K are given by

(4) $$Y\left(U\right)=j_{0}\frac{2U/\Gamma }{1+{\left(U/\Gamma \right)}^{2}},\;K\left(U\right)=\frac{2j_{0}}{1+{\left(U/\Gamma \right)}^{2}}$$

The dephasing $\Gamma =\hbar /\tau =U_{c}\;$defines the region of negative differential resistance. It can be calculated by NEGF [29] or extracted from static current-voltage measurements [48]. The peak current density is$\;j_{0}$. Ideal superlattices would have a perfectly antisymmetric I–V [7]. However, measured I–V. can be strongly different from this idealized case and among other possible reasons for this we can point out the fact that the interfaces of GaAs over AlAs are less sharp than those of AlAs over GaAs [29,43]. In the calculations shown here, this effect is described by the current flow asymmetry parameter $\delta =\Gamma^{+}/\Gamma^{-}=j_{0}^{+}/j_{0}^{-}$, is used [30,31,44]. Therefore, as δ deviates from the ideal case, δ=1, even harmonics buildup and other interesting effects develop, as described next. An Ansatz correction to Equation (4) successfully described this effect [29],

(5) $$j_{0}=\{\begin{array}{c} j_{0}^{-},\;\;U<0\\ j_{0}^{+},\;\;U\geq 0 \end{array},\;\Gamma =\{\begin{array}{c} \Gamma^{-},\;\;U<0\\ \Gamma^{+},\;\;U\geq 0. \end{array}$$

This Ansatz explains the experimentally detected even harmonic generation in Refs. [29,43,48] but the discontinuity at the origin leads to numerical instabilities and a noisy output for low incident powers, which is hard to distinguish from experimental noise. This problem is solved by introducing a New Ansatz

(6) $$\Gamma =\Gamma^{-}+\left(\Gamma^{+}-\Gamma^{-}\right)F\left(U/\Gamma^{+}\right),\;j_{0}=j_{0}^{-}+\left(j_{0}^{+}-j_{0}^{-}\right)F\left(U/\Gamma^{+}\right).$$

where $F\left(x\right)=\frac{1}{1+e^{-kx}}$ denotes the logistic function. For the results presented in this paper, the choice is k=20.

At this point, a brief discussion about the origin of the “imperfect I–V.” is in order. The measured I–V curves deviate from the ideal Ezaki-Tsu model ($\Gamma^{+}=\Gamma^{-},\;\;j_{0}^{+}=j_{0}^{-}$). To understand microscopically why $\Gamma^{+}\neq \Gamma^{-},\;\;j_{0}^{+}\neq \;j_{0}^{-}$), let us remember that typical modeling in the literature is based on ideal superlattices with infinitely sharp structures. This, of course, is not true and during epitaxial growth, AlAs sits better over GaAs than vice versa. We call the growth direction the z-direction. What happens is that if they have either positive or negative bias, the current density flows in different directions so the scattering from GaAs to AlAs is different than from AlAs to GaAs.

We have successfully modeled the effect by means of an interface roughness selfenergy, $\Sigma_{\alpha ,\beta }^{≶}\left(k,E\right)$. We express the electronic states in terms of Wannier states $\psi_{\alpha }\left(z\right)$, by solving the system Hamiltonian and find the NEGF Green’s functions $G_{\alpha ,\beta }^{≶}\left(k,E\right)$, the corresponding selfenergy within the second Born approximation is

(7) $$\Sigma_{\alpha ,\beta }^{≶}\left(k,E\right)=\eta^{2}\underset{\gamma ,\lambda ,j}{\sum }{\left(\Delta E_{j}\right)}^{2}\int d^{2}r\;e^{i\left(k-k^{\prime }\right)\cdot r}e^{-\frac{r}{\lambda }}G_{\gamma ,\lambda }^{≶}\left(k^{\prime },E\right)\psi_{\alpha }^{*}\left(z_{j}\right)\psi_{\gamma }\left(z_{j}\right)\psi_{\lambda }^{*}\left(z_{j}\right)\psi_{\beta }\left(z_{j}\right)$$

Here, $\psi_{\beta }\left(z_{j}\right)$ is the Wannier state *β* at interface *j* and $\Delta E_{j\;}$is the intersubband offset. We have obtained excellent comparisons between theory and experiments in Ref. [29] by choosing $\Delta E_{j\;}$ = −1 eV, *η* = 0.1 and *λ* = 5 nm for the GaAs over AlAs interface and $\Delta E_{j\;}$ = −1 eV, *η* = 0.2 and *λ* = 5 nm for the AlAs over GaAs interface.

## 3. Results and Discussion

The results discussed next are for GaAs-AlAs superlattices, characterized by a period *d* = 6.23 nm. The main input parameters for Equations (3)–(6) are: $j_{0}^{+}=2.14\times 10^{9}\;A/m^{2}$, $\Gamma^{+}$ = 21 meV, from Ref. [29].

Before showing numerical results, it is important to further highlight that the nonlinear response is ruled by the parameter $\alpha =eE_{ac}d/\left(h\nu \right)$. Thus, the period plays a major role and the larger the period, the smaller the input power needed to achieve higher harmonic power output. However, the period cannot be too large, or tunneling is reduced and the superlattice behaves like a series of isolated Quantum Wells. There is thus an interplay between sufficiently high tunneling for high current and the power needed to achieve large α with corresponding high harmonic power output.

Figure 1, Figure 2 and Figure 3 show the combined effect of input power and current flow asymmetry on the resulting peak output power asymmetry of the second harmonic, as the bias is swept from negative to positive values.

As discussed in the Section “Materials and Methods”, the asymmetry in the power output on the right and left sides is ruled by the parameter $\delta =\Gamma^{+}/\Gamma^{-}=j_{0}^{+}/j_{0}^{-}$. In all curves in Figure 1, we have fixed $\Gamma^{+}$ and $j_{0}^{+}$. Thus, the peak output on the right side is the same, while on the left it changes according to the value of *δ* used. The black line is for *δ* = 1, i.e., symmetric current flow leading to perfectly antisymmetric I–V. The blue and green curves are, respectively, for *δ* = 1.1 and *δ* = 0.91. Consistently with the I–V. asymmetry breaking seen in the inset, δ>1 leads to a higher peak on the left compared to the right and vice-versa δ<1. Note that there is a complex interplay of power input, asymmetry parameter and applied voltage range controlling the actual output power. The changes from left to right are not always very large, but they are sufficient to explain the asymmetry seen experimentally in Ref. [33]. The next two figures show in more detail the evolution of the asymmetry in peak output for increasing powers of the input oscillating field.

Figure 1 shows that in order to obtain the asymmetry in maxima for negative and positive bias found in Ref. [33] and unexplained by their simulations, we need to consider an asymmetry in the current flow, i.e., δ≠1. The peak moves from right to left depending on the corresponding maxima/minima in I–V., which is given for reference in the inset. Figure 2 and Figure 3 illustrate the evolution of the second harmonic generation as the amplitude of the input oscillating field increases, following the experimental results in Ref. [33] closely. In other words, the maxima for positive or negative biases are different. In contrast, the simulations in Ref. [33] have a fully symmetric output, since they correspond, in our approach, to δ=1. Figure 4 shows that the Old Ansatz of Equation (5) is safe to use for sufficiently high input powers of the oscillating input field, but fails for low powers, while the New Ansatz of Equation (6) remains numerically stable due to continuity at the origin in contrast to Equation (5). This can make a large difference when analyzing experiments with usual low excitation powers. In more detail, Equation (5) has a step function Ansatz, which is discontinuous for U=0. This leads to numerical errors for small voltage. If we look at small changes in the current and take the limits U→0 and U →∞, we see that for low voltage, there is a linear increase with U proportional to (δ−1). For large voltage, there is a fast 1/U decrease, also proportional to (δ−1). Of course, the errors disappear for (δ=1). This means that the output is more sensitive to variations in the range where the output is small, corresponding to low input power (or small α) and relatively insensitive at large voltages and large α. In Figure 4b the output is so large and grows so fast with the increasing voltage that correspondingly small fluctuations are not visible around U=0. The noise at low voltage and low power is a purely numerical effect. Per coincidence, experiments also show similar noise under the same conditions, which are likewise due to the fact that experimental errors and detection scheme limitations are larger in this range and fade away for sufficiently high powers. See Figure 3 of Ref. [48], where experimental results for high order harmonics are compared and contrasted with a theory that uses the step function discontinuous Ansatz of Equation (5).

In summary, this paper shows relevant progress in the study of harmonic generation in semiconductor superlattices with a focus on two important results: (i) A more efficient Ansatz solution for asymmetric current flow. This eliminates numerical artifacts of a previous approach and delivers a robust and efficient analytical approach to the problem. (ii) A concrete explanation for the previously unexplained asymmetric power output found in the literature. This happens under combined static bias and oscillating field, as the bias is swept from negative to positive values. This theory has the potential to further the understanding of new nonlinear phenomena and to be used as a powerful design tool for new GHz-THz devices.

## Data Availability

All data that support the findings of this study are present in the paper. Additional data related to this paper may be requested from the author upon reasonable request. Code availability-The code that was used to simulate the findings of this study is available from the corresponding authors upon reasonable request.
